# Combined Effect of Platelet-Rich Fibrin Matrix (PRFM) and Demineralized Freeze-Dried Bone Allograft (DFDBA) in Immediate Implant Placement: A Single-Arm Clinical Trial

**DOI:** 10.7759/cureus.29728

**Published:** 2022-09-29

**Authors:** Komal R Bhombe, Pavan Bajaj, Bhushan Mundada, Prasad Dhadse, Chitrika Subhadarsanee, Ranu R Oza

**Affiliations:** 1 Department of Periodontology and Implantology, Sharad Pawar Dental College and Hospital, Datta Meghe Institute of Medical Sciences, Wardha, IND; 2 Department of Oral and Maxillofacial Surgery, Sharad Pawar Dental College and Hospital, Datta Meghe Institute of Medical Sciences, Wardha, IND

**Keywords:** demineralized freeze-dried bone allograft (dfdba), platelet-rich fibrin matrix (prfm), jumping distance, bone graft, dental implants, immediate implant

## Abstract

Introduction

Placement of immediate implants in contrast to delayed implant placement may be favorable. The factors contributing to this are shortened overall treatment time, aid in ideal orientation and fixture placement, bone preservation following extraction, and achieving optimal aesthetics involving soft tissue. However, the gap distance between the surface of the implant and the buccal bony wall during implant placement is critical for subsequent bone healing in a fresh extraction socket. Considering that as the gap broadens, the amount of bone-to-implant contact (BIC) decreases, causing an apical shift of the highest bone-implant contact. Incorporating a bone substitute material (BSM) within the fixture-socket gap preserves alveolar ridge volume by minimizing socket remodeling and encouraging de-novo bone formation.

Aim and objectives

To evaluate the efficacy of platelet-rich fibrin matrix (PRFM) and demineralized freeze-dried bone allograft (DFDBA) in fresh extraction socket with simultaneous implant placement.

Methods

Implants were immediately placed in 12 patients following a two-stage submerged protocol. The combination of PRFM and DFDBA was used to fill the gap between the implant body and the surrounding socket wall. The final restoration was placed after 3 months following implant placement. The full mouth plaque, gingival bleeding index, and gingival esthetics scores were assessed at baseline, 3, and 6 months. The crestal changes were evaluated using intraoral periapical radiographs (IOPA) at baseline, 3, and 6 months. Cone beam computed tomography (CBCT) images were obtained at baseline and 6 months after implant loading to analyze the buccolingual changes.

Results

At 6 months follow-up, the coronal bone remodeling detected on CBCT revealed a minimal (0.1 mm) narrowing of the alveolar ridge in a buccolingual direction, with a mean bone loss of 0.10+0.09, which was statistically non-significant (p > 0.05). Implant success was 100% at 6 months after loading as determined by Akbrektsson’s criteria for implant success.

Conclusions

The adjunctive use of PRFM with DFDBA following immediate implant placement yielded a significant reduction in bone resorption and maintenance of buccolingual dimensions.

## Introduction

The placement of oral implants immediately following extraction was initially described by William Schulte and Heimke in 1976 [1]. According to a study conducted by Lazzara, immediately placed implants helped to preserve the dimensions of extraction sockets in humans [2]. Numerous prospective and retrospective studies have noted high survival rates of immediate implants [3-10]. In a consensus statement, Hammerle et al. (2004) recognized that immediate implants belonged to the type I procedure, i.e., placement of implant immediately following tooth extraction and belonging to the same surgical procedure, which in contrast to delayed implant placement may be favorable considering the shortened overall treatment time owing to the minimum number of surgical procedures and optimal availability of existing bone to allow for primary stability of the implant [11]. Moreover, it has been proposed to aid in ideal orientation and placement of the fixture, preservation of the bone after extraction, and achieving a good amount of aesthetics involving soft tissue [12]. However, predominantly soft cancellous bone of the fresh extraction socket, collectively with the high aesthetic demands of the patients, bring up several challenges when planning successful placement and prosthetic rehabilitation of the immediate implant.

Following tooth extraction, the sockets often exhibit dimensions that may be significantly greater than the standard implant diameters. Consequently, peri-implant voids often exist due to the presence of a gap between the alveolus and the implant fixture. The space thus identified is called horizontal gap distance or jumping distance [13]. The rough surface implants have demonstrated spontaneous bone repair and osseointegration when possessing a horizontal distance (HD) of 2 mm or less. HDs > 2 mm have been known to possess unpredictable bone healing. Thus, the consensus became that horizontal gap distances of >1.5- 2 mm most likely require the placement of particulate bone substitute materials (BSM), including allograft/xenograft covered by a membrane for prevention of soft tissue in-growth, thus encouraging the osteogenic cells to engage in the bone regeneration [12].

Numerous studies have suggested incorporating a BSM within the gap distance to preserve the socket volume by minimizing socket remodeling and encouraging new bone formation [14-16]. The osteoinductive potential of demineralized freeze-dried bone allograft (DFDBA) is associated with the presence of bone morphogenetic proteins (BMPs); it contains BMP 2, 4, and 7, which help to stimulate osteoinduction [17]. DFDBA tends to degrade more rapidly, enabling new bone formation [18]. In essence, the overall advantage of allografts is that it has mechanical properties comparable to autogenous bone and may have the collagenous matrix and proteins found in natural bone, despite the fact that it lacks vital cells. Similarly, the handling characteristics are equivalent to the autologous bone, albeit the shorter surgical time required for implantation and their increased availability are apparent advantages over autogenous bone [19].

A novel preparation of platelet concentrate known as platelet-rich fibrin matrix (PRFM) has recently been studied in various intraoral and extraoral procedures. The mechanical properties of PRFM translate it into a biological matrix that is easy to manipulate and implant in a variety of tissue repair and regenerative procedures [20]. It has been demonstrated that the vital platelets in PRFM generated six growth factors, including platelet-derived growth factor (PDGF), vascular endothelial growth factor (VEGF), fibroblast growth factor (FGF), epidermal growth factor (EGF), transforming growth factor (TGF), and insulin-like growth factor (IGF) in about the sustained concentration for a period of 7 days [21]. In contrast, BMPs act principally at the later stages of osteoinduction, mainly during mesenchymal cell differentiation and vascular proliferation. PRF entraps circulating stem cells, resulting in superior healing of large osseous defects where there is a differentiation of migrating stem cells into the osteoblast phenotype [22]. When combining graft materials such as DFDBA with platelet concentrates like PRF enables, these two separate wound healing processes to take place concurrently. Thus, a combination of platelet concentrates and DFDBA may promote bone regeneration and enhance the biological activity of the graft material [23]. Hence, the present study was undertaken to evaluate crestal bone changes around immediately placed implant in a fresh extraction socket with simultaneous placement of DFDBA and PRFM using a two-stage protocol.

## Materials and methods

Study design

This single-arm clinical trial was conducted at the Department of Periodontics, Faculty of Dentistry, Sharad Pawar Dental College, Datta Meghe Institute of Medical Sciences, Maharashtra, India. Institutional Ethical Committee approved the study protocol, DMIMS (DU) (DMIMS (DU)/IEC/Aug-2019/8270, dated 04/09/2019). The project was funded by Indian Council Medical Research (ICMR) (letter No. 3/2/June-2020 I PG-Thesis-HRD (47D), Dated: 02/09/2020) and registered in Clinical Trial Registry-India (CTRI) with registration number: CTRI/2020/11/028974.

Sample size calculation

OpenERP software, Version 3, open-source calculator - SSMean was used to calculate the sample size. Crestal bone loss was the variable of interest in this study. Sample size calculation was done with a 95% confidence interval, the power of the test being 80%. Input data revealed the ratio of sample size is restricted to 1. The statistical approach used was paired t-tests. The calculation revealed that a sample size of 12 patients was required.

Patient recruitment

Patients were recruited from the outpatient department of periodontics at Sharad Pawar Dental College, DMIMS (DU), who required single tooth extraction and were willing for fixed immediate replacement with implants. After the clinical and radiographic evaluation, 12 patients precisely met the following inclusion criteria: 1) tooth requiring extraction due to root fractures, endodontic failures, internal and external resorption, over-retained deciduous teeth, non-restorable carious lesions, residual roots; 2) good oral hygiene is defined as a full mouth plaque score ≤ 25%; 3) presence of opposing natural tooth; 4) presence of adjacent teeth; 5) thick gingival biotype; 6) radiographic and clinical appearance of intact alveolar bony walls; 7) presence of at least 4 mm of bone beyond the root apex; 8) D-1 or D-2 bone quality.

Exclusion criteria were: 1) compromised general health conditions that would jeopardize the bone healing process, e.g., diabetes, osteoporosis, blood disorders, and allergies; 2) severe maxilla-mandibular space discrepancies; 3) para functional habits (bruxism or clenching); 4) teeth with interdental spacing/proclined teeth/rotated or mal-aligned anterior teeth; 5) history of alcoholism, excessive smoking, or drug abuse; 6) D-3 and D-4 bone quality; 7) width of keratinized gingiva is less than 2 mm at the implant site; 8) debilitating temporomandibular joint pathology; 9) untreated dental diseases; 10) pregnant and lactating mothers; 11) history of radiotherapy and chemotherapy.

Presurgical Phase

**Figure 1:**
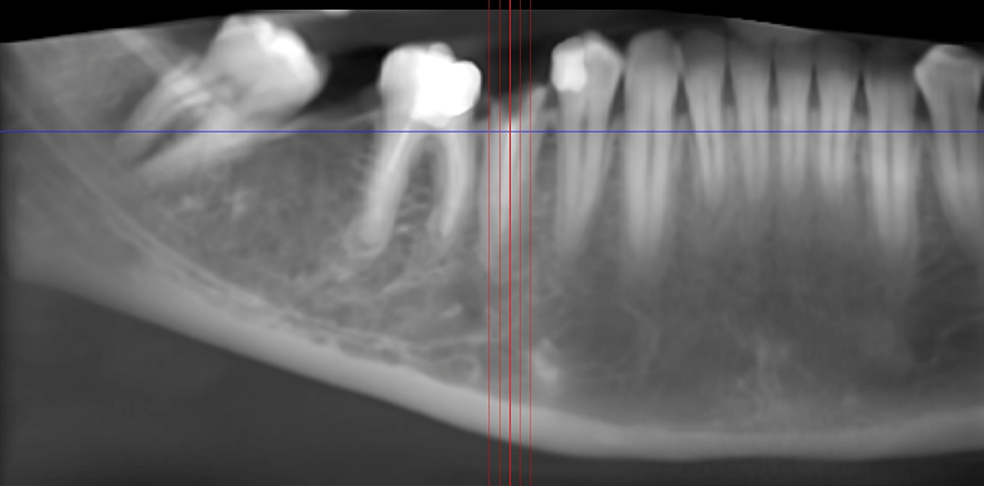
CBCT depicting panoramic view Panoramic view showing root piece with lower right second premolar. CBCT: cone beam computed tomography

**Figure 2:**
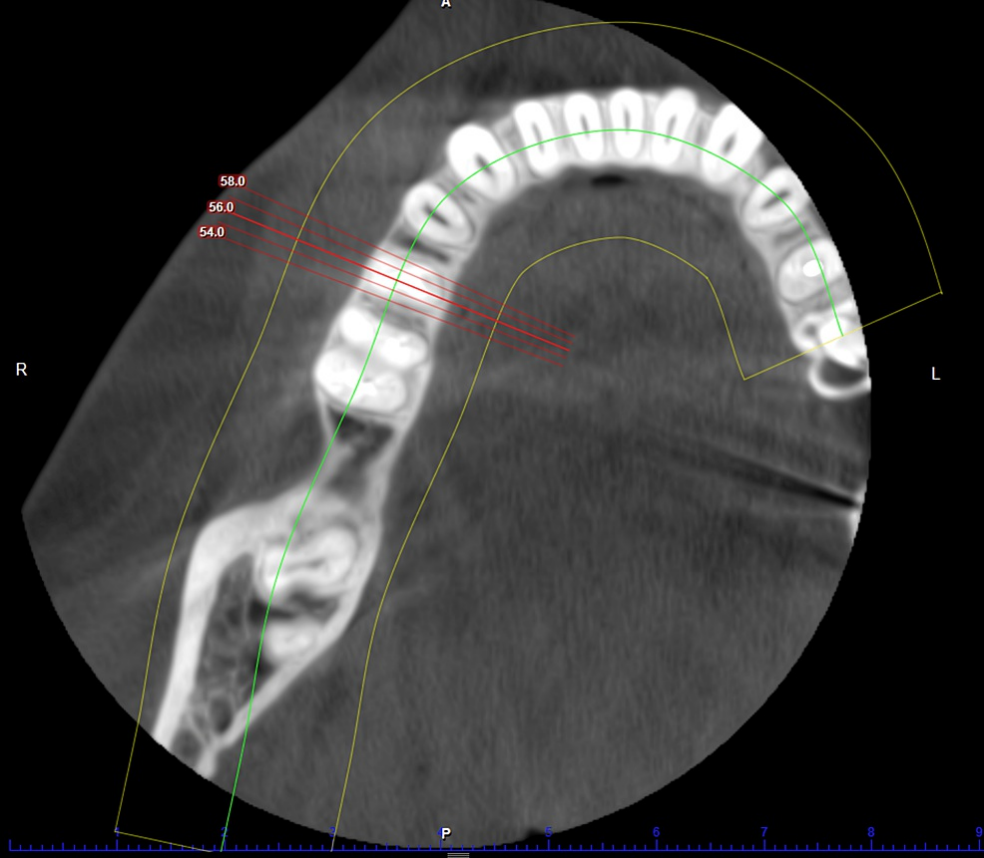
CBCT depicting axial view Axial view representing buccolingual alveolar ridge width with respect to lower right second premolar. CBCT: cone beam computed tomography

**Figure 3:**
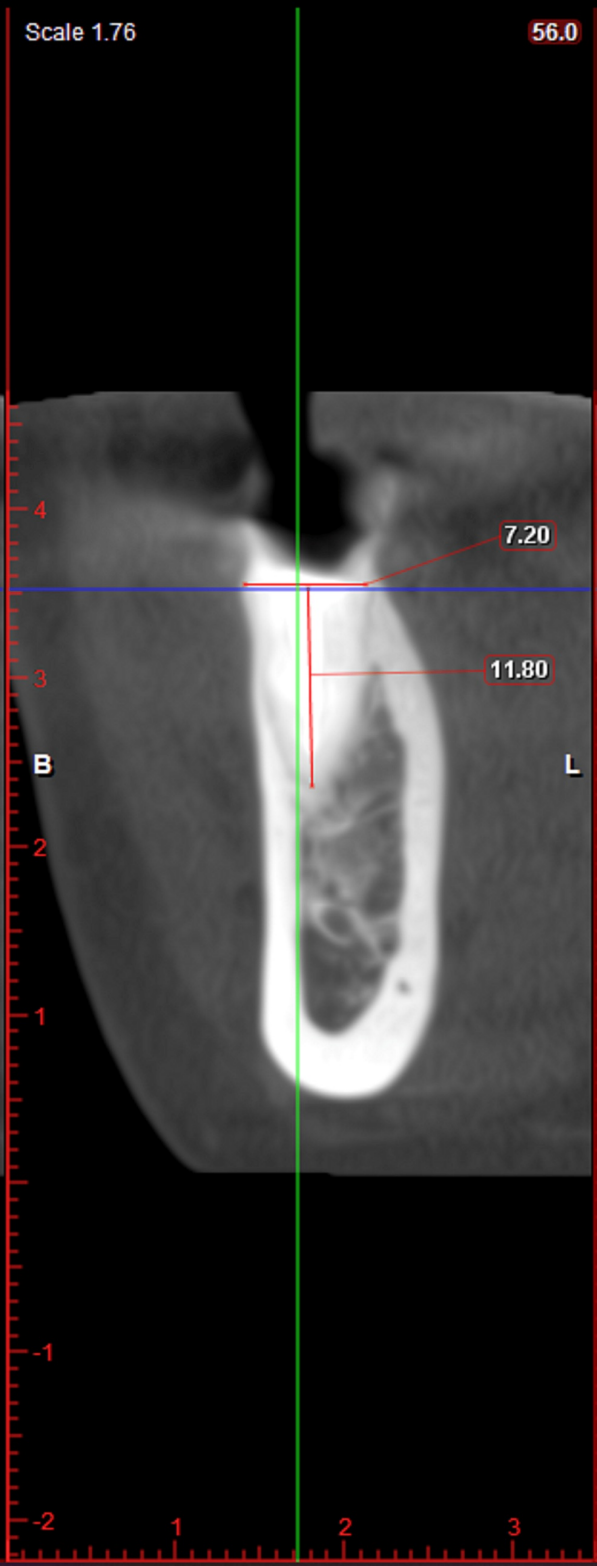
CBCT showing coronal view Coronal view representing apico-coronal dimensions of root piece with lower right second premolar. CBCT: cone beam computed tomography

After complete examination and diagnosis, initial therapy consisting of oral hygiene instructions, supragingival, and subgingival scaling was performed. Plaque control instructions were repeated until the patient achieved a plaque score of ≤ 1. Prior to the surgical phase, a diagnostic cast of each patient was prepared to establish the maxilla-mandibular relationship. The clinical photographs, periapical radiographs, and cone beam computed tomography (CBCT) were obtained for all the patients. An intraoral periapical radiograph (IOPA) was taken at each implant site with a long cone (XCP Rinn, Dentsply) paralleling technique at baseline, 3 months, and 6 months. Radiographic measurements were obtained by utilizing a film mount with a Milli meter grid scale (Nix Company Ltd. Tokyo, Japan). These are pocket mount-type grids for the intraoral film. Grid-scale lines are printed at 1 mm intervals with bold lines at 5 mm intervals, which were used to measure the vertical and horizontal crestal bone level around the implants. The overall advantage of using CBCT in implant dentistry is related to its ability to acquire detailed volumetric image data of the maxillofacial region for diagnostic and presurgical planning purposes [24]. Figures 1-3 depict the preoperative CBCT evaluation.

Clinical indices were recorded at baseline (before surgery), 3 months after the second surgery, and 6 months (i.e., 3 months after final prosthesis). The patient’s oral hygiene status was evaluated by full mouth plaque index (FMPI) as an expression of the level of full mouth supragingival plaque accumulation using Turesky-Gilmore-Glickman Modification of Quigley Hein (1970). Gingival inflammation was assessed by full mouth papillary bleeding index (FMPBI) by Muhlemann H. R. (1977). Measurement of implant stability using Misch’s clinical implant mobility scale (CIMS) at 3 months after definite prosthesis for all patients was carried out. The CIMS scale used was as follows: 0: absence of clinical mobility with 500 g in any direction; 1: slight detectable horizontal movement; 2: moderate visible horizontal mobility up to 0.5 mm; 3: severe horizontal movement greater than 0.5 mm, and 4: visible moderate to severe horizontal and any visible vertical movement.

Surgical Procedure

**Figure 4:**
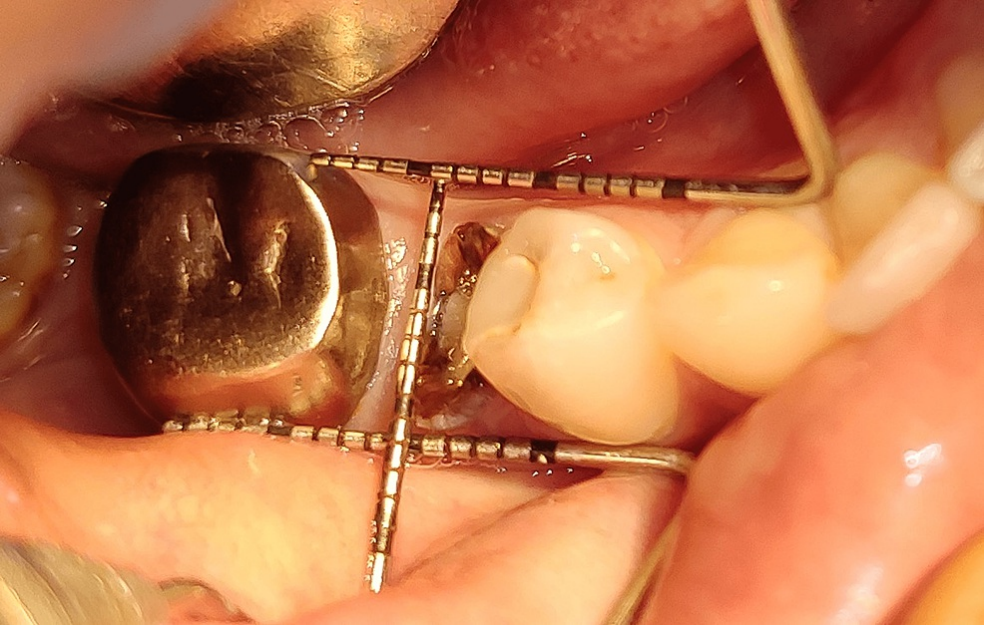
Preoperative view Image depicting clinical buccolingual and mesiodistal dimensions.

**Figure 5:**
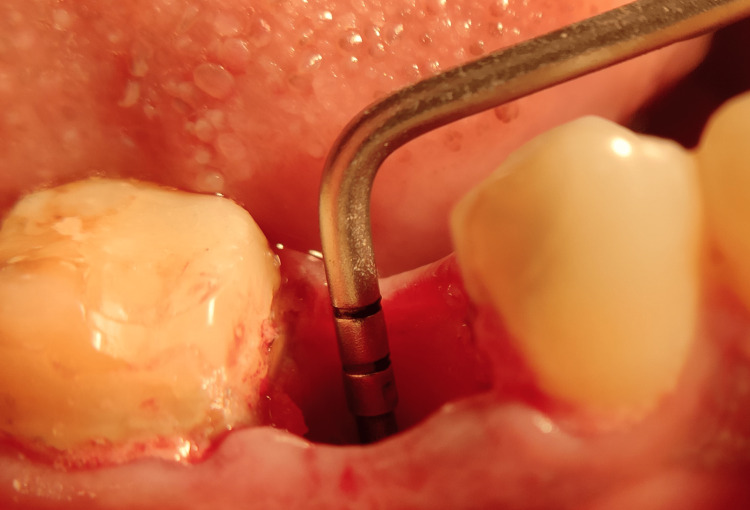
Post-extraction view Image depicting socket depth post-extraction.

Atraumatic extraction: The surgical protocol was followed with complete asepsis and infection control. Briefly, after induction of local anesthesia, a sulcular incision was performed on the buccal and lingual aspects of the teeth to be removed. Full-thickness mucoperiosteal envelope flaps were reflected on buccal and lingual aspects so as to visualize the bone plates. The initial incision no. 15 or 12 bard parker surgical blade along with separation of the supra-crestal gingival fibers, followed by the separation of the periodontal ligament at the mesial and distal aspect of the root piece. To preserve the buccal plate, the periodontal ligament fibers were further separated with the use of periotomes, and the teeth were gently luxated and removed. Figures 4-5 depict the preoperative and post-extraction view respectively.

**Figure 6:**
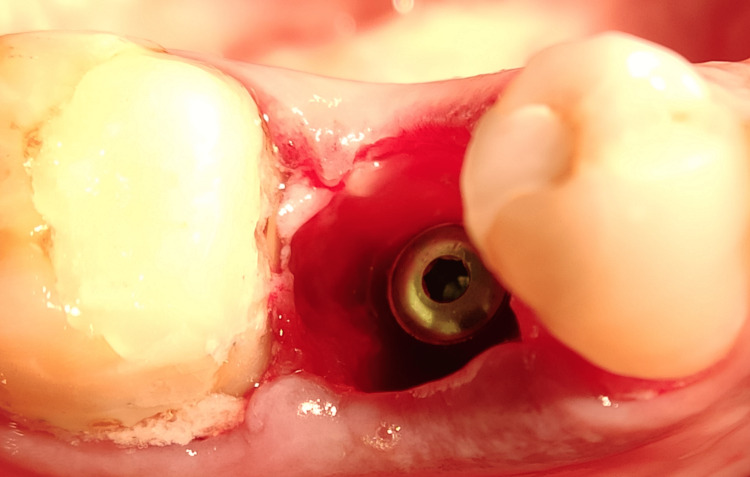
Fixture in place Image depicting dental implant placed in position with lower right second premolar.

Fixture placement: Once the tooth was extracted, the sockets were examined for any fracture of the walls of the socket, and then it was thoroughly debrided, removing all the visible granulation tissue. The preoperative classification proposed by Salama and Salama (1993) was used to categorize the cases, and only type I extraction sites (four-wall sockets) were selected [25]. A sequential drilling protocol for osteotomy was carried out using OSSTEM TS III KIT with drill sequences of 2 mm, 3 mm, 3.5 mm, 4 mm, 4.5 mm, and 5 mm, depending on the diameter of implant and speed ranging from 500 to 1200 rpm under copious irrigation. The drill was extended 3-4 mm beyond the apex of the socket to ensure primary stability after placement taking care of anatomical boundaries. Once the osteotomy site was prepared, the largest and widest possible implants were placed. Selected implants were two-piece with corkscrew threaded design, platform switched implants having morse taper connection. The implant surface was sandblasted with alumina and acid etched as selected from Osstem TS III dental implant system. The implants of length 10-13 mm and diameter of 3.5-5 mm were placed by motor insertion with an insertion speed of 15 rpm at the recipient site. this protocol was followed uniformly for all the implants in the study. Once the implant was in place, the HD between the implant body and the inner surface of the buccal wall was measured, and only those implants that had a gap of 1.5-2 mm or more were included in the study. The insertion torque for all implants ranged between 25 and 40 Ncm. All implants were checked for mobility with the two blunt ends of the instrument for any perceivable mobility. The primary stability can be easily confirmed at the time of insertion of the cover screw into the implant. Figure 6 depicts the fixture placement post-extraction.

**Figure 7:**
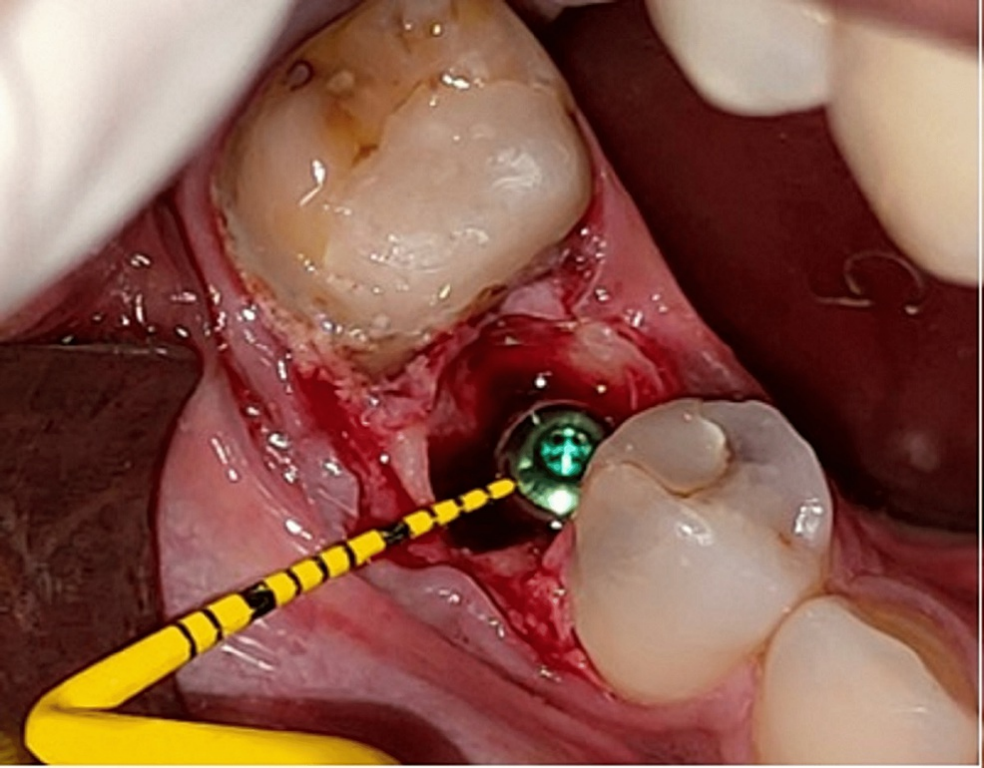
Depicting jumping gap distance

**Figure 8:**
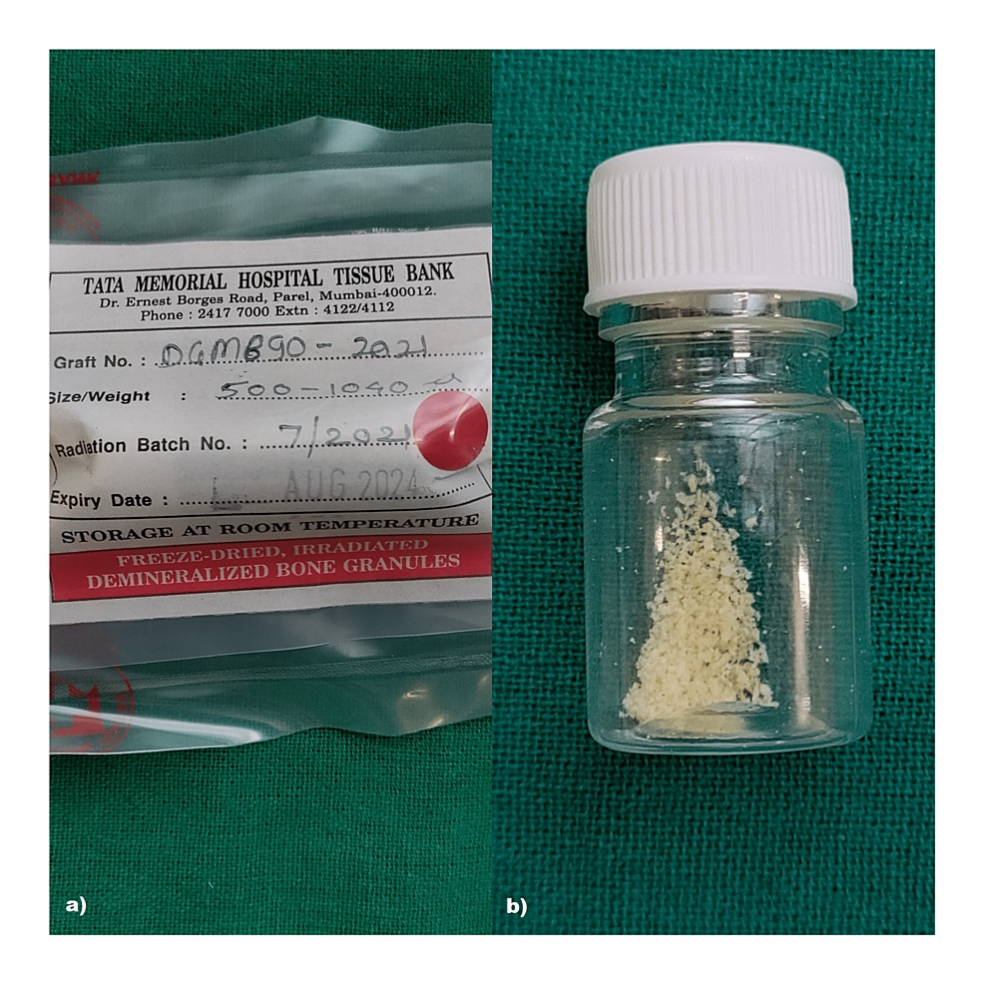
Depicting DFDBA as bone substitute material a) DFDBA obtained from Tata Memorial Hospital Tissue Bank; b) particulate DFDBA (Size: 500-1040 μ) DFDBA: demineralized freeze-dried bone allograft

**Figure 9:**
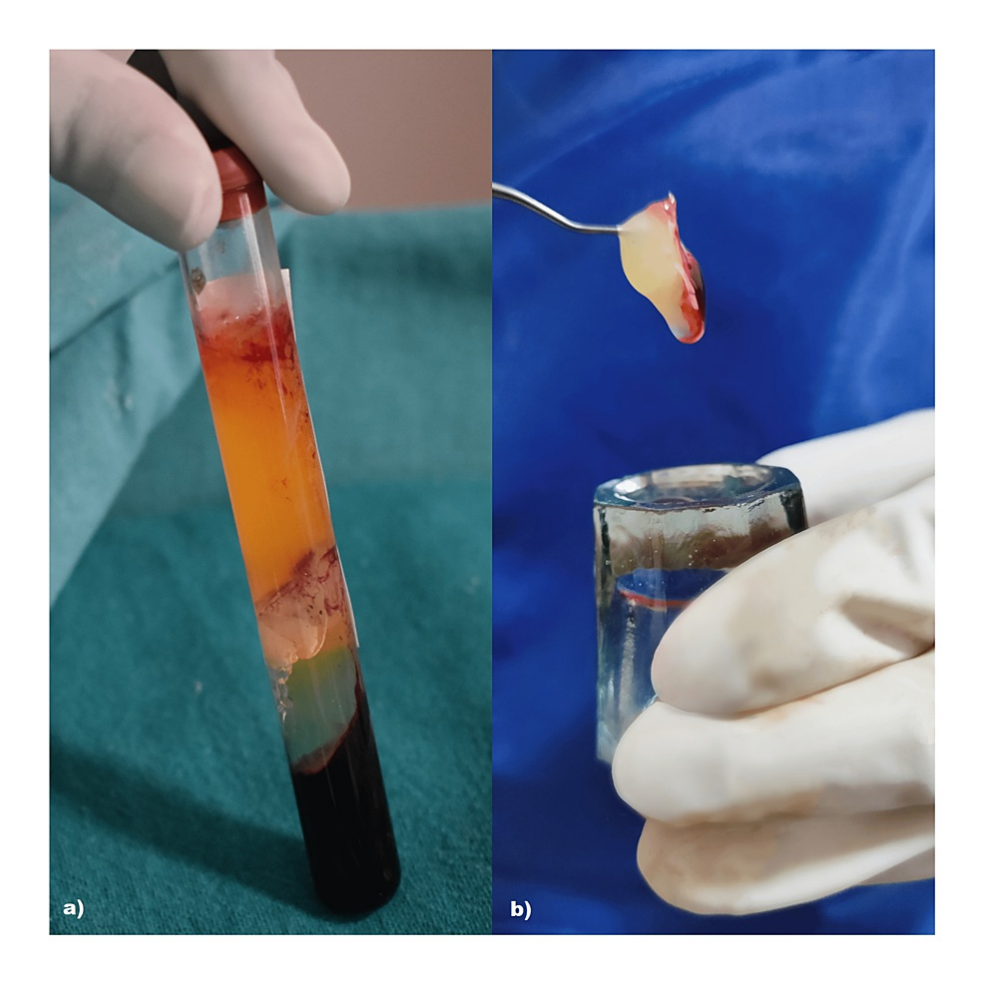
Obtained platelet-rich fibrin matrix a) PRFM test tube after centrifugation; b) PRFM clot obtained PRFM: platelet-rich fibrin matrix

**Figure 10:**
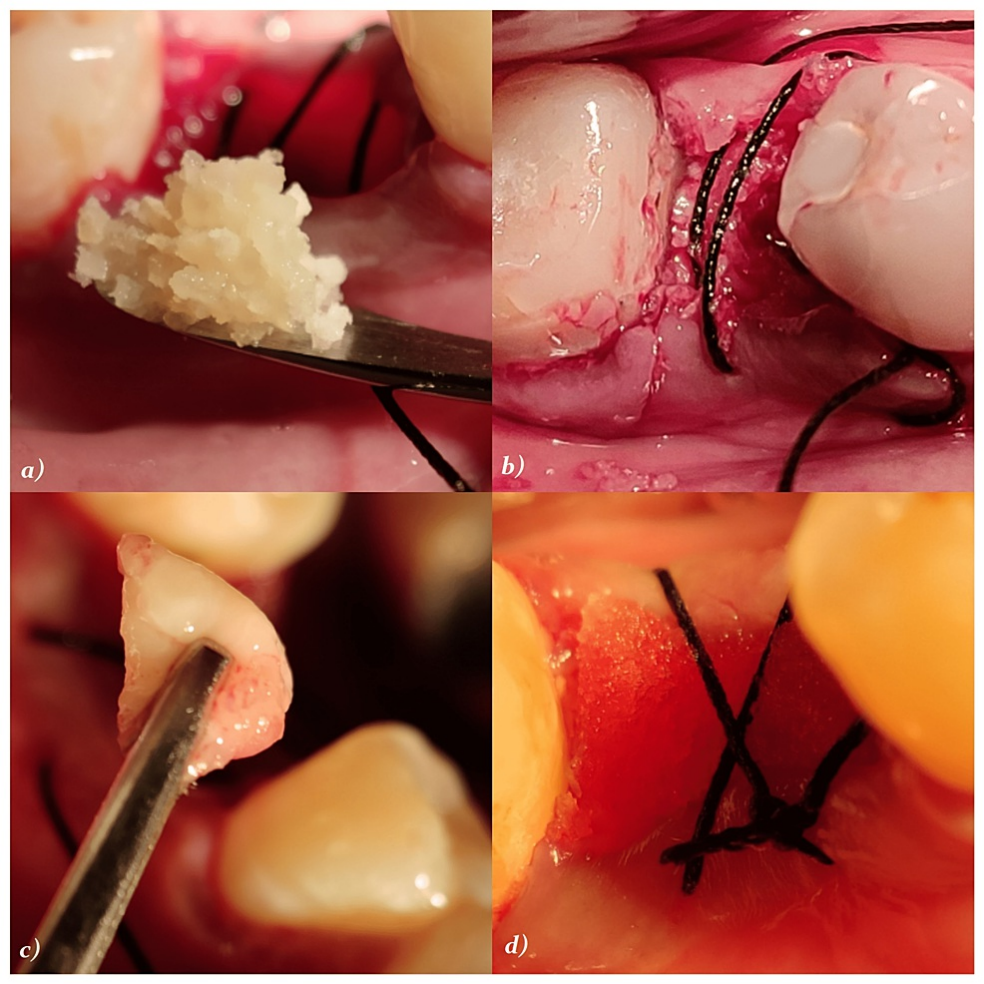
Depicting DFDBA and PRFM placement around the implant a) DFDBA placement; b) bone graft in place around the implant; c) placement of PRFM membrane; d) sutures in place DFDBA: demineralized freeze-dried bone allograft; PRFM: platelet-rich fibrin matrix

Management of jumping gap distance: All the circumferential bone defects or horizontal defects of >1.5-2 mm were augmented using DFDBA particles (Tata Memorial Hospital, Tissue Bank Mumbai, India) along with PRFM (MERISIS PRFM, DiponEd Bio intelligence LLP, Bangalore, Karnataka, India) were in place in the extraction site to cover the thin bone. The preparation protocol of PRFM included the use of 13 ml MERISIS PRFM tube with a gel separator and blood-loading capacity of 10 ml. It contains 1 ml of 0.1% gluconate as an activator of PRFM. 10 ml of blood sample was collected within 1 min from the antecubital vein of all the patients. The samples were immediately transferred within 30 seconds in the MERISIS PRFM and placed in centrifugation machine. It was centrifuged at rpm of 3000 for 10 min using single-spin centrifugation. The supernatant was obtained and the top of the gel was removed through syringe and the clot obtained was then used. The material was intimately packed, especially on the buccal aspect, followed by implant placement. Finally, after stabilization, the buccal flap was positioned around the implant and sutured to the lingual/palatal flap using a simple interrupted suture (3-0, 2 metric non-absorbable braided silk surgical suture). Figure 7 depicts the jumping gap distance. Figures 8-10 depict DFDBA and PRFM used and their placement at the implant site respectively.

Postoperative Care

**Figure 11:**
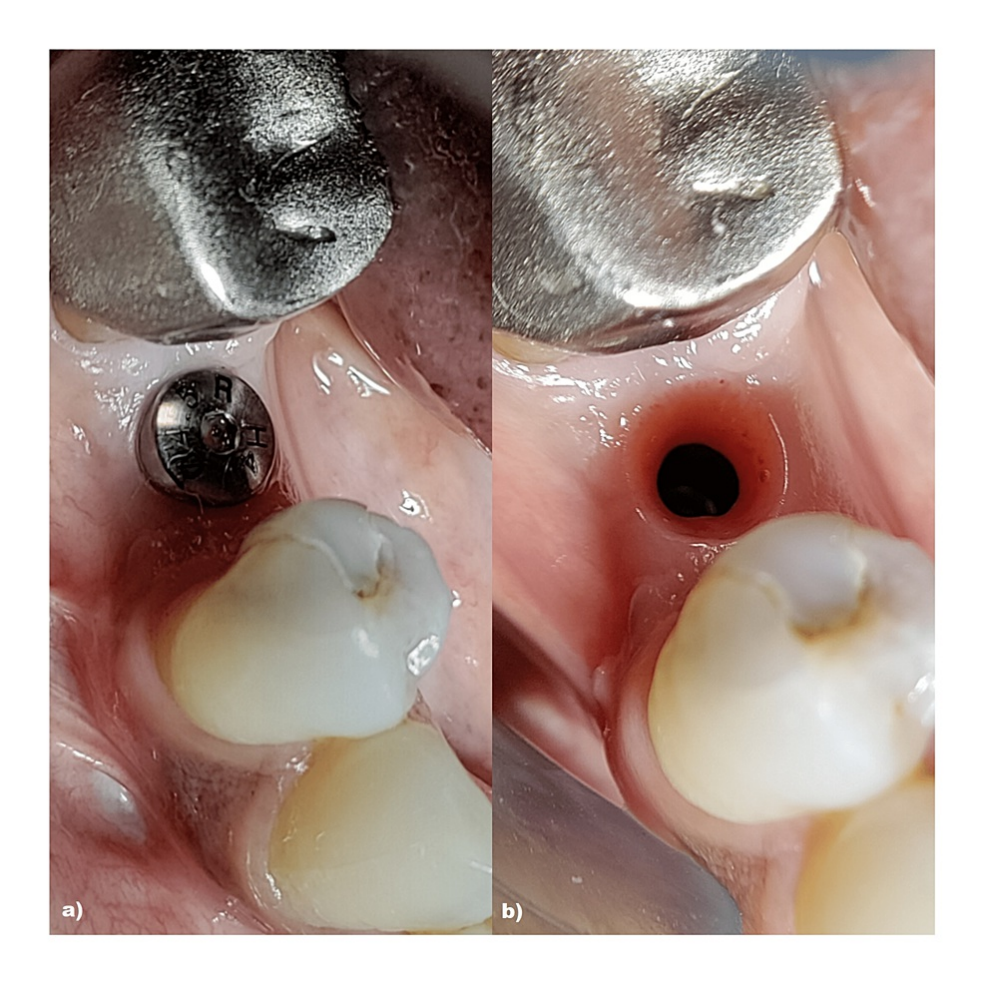
Depicting second-stage surgical procedure a) Placement of gingival former; b) gingival collar obtained after healing

**Figure 12:**
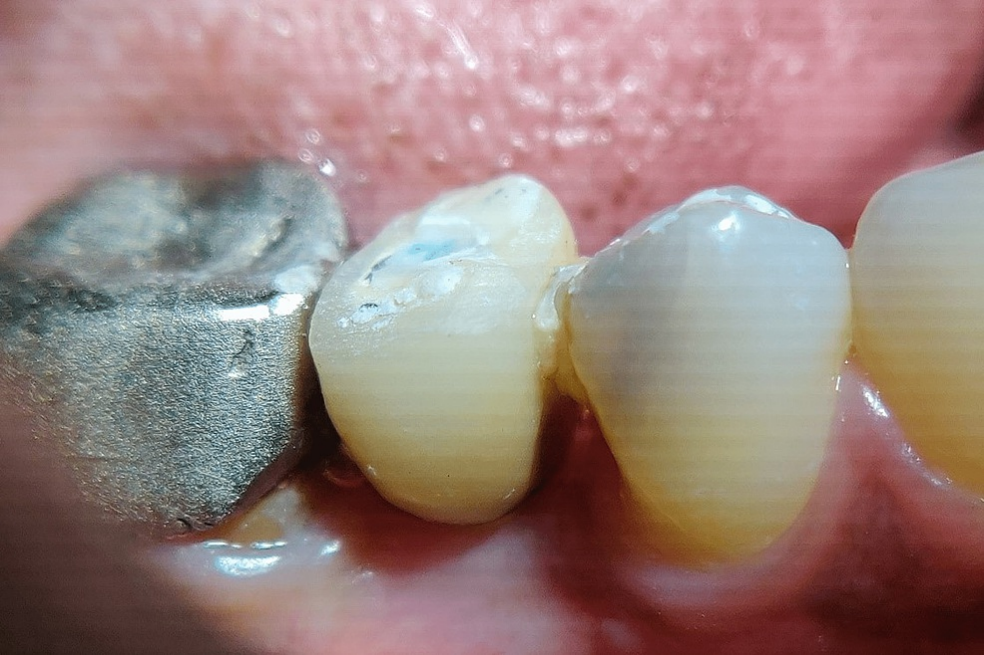
Depicting definitive prosthesis after 3 months

All the patients received antibiotics (Cap amoxicillin, 500 mg t.i.d., Tab metronidazole 250 mg t.i.d) and analgesics (Tab ibuprofen 200 mg t.i.d.) after surgery for a period of 5 days. Patients were instructed not to brush in the treated area for 3 days following surgery. A chlorhexidine di-gluconate rinse (0.12%) was advocated twice daily, preferably for 1 minute up to 7 days. Sutures were removed after 7 to 10 days of implantation, and patients were assessed for any postoperative pain, discomfort, or presence of suppuration from the implant site. The second-stage flapless surgery was performed 3 months after implant placement. The implants were uncovered by a tissue punch and a gingival former was connected to allow guided soft tissue healing for 8-10 days followed by placement of definitive prosthesis. Figures 11-12 depict the gingival collar obtained and definitive prosthesis placement.

Clinical and radiographic parameters

Clinical indices were recorded at baseline (before surgery), 3 months after the second surgery, and 6 months (i.e., 3 months after final prosthesis). Recording of clinical indices was carried out by the operator in all the patients. Mean of these values were obtained for the assessment of the result. The patient’s oral hygiene status was evaluated by FMPI as an expression of the level of full mouth supragingival plaque accumulation using Turesky-Gilmore-Glickman Modification of Quigley Hein (1970). Gingival inflammation was assessed by FMPBI by Muhlemann H. R. (1977).

CBCT was carried out for presurgical diagnosis and evaluation of the volumetric status of available bone to guide the implant placement. Buccolingual diameter of alveolar ridge (mid-buccal) before the extraction and following 6 months of implant placement was measured using CBCT.

Radiographic measurements were obtained by utilizing a film mount with millimeter grid scale (Nix Company Ltd. Tokyo, Japan). These are pocket mount-type grids for intraoral film. Grid-scale lines are printed at 1 mm intervals with bold lines at 5 mm intervals. The developed IOPA films were inserted in the mount to assess the changes in the interproximal alveolar crestal bone height. To determine the vertical radiographic bone level, the distance from the implant shoulder to the most coronal bone in contact with implant (DIB) and radiographic HD between the implant to the crest of the alveolar bone was measured. All measurements were carried out on both the mesial and distal aspects of each implant and expressed in millimeters. For each implant, mean vertical and horizontal bone loss was calculated on the mesial and distal surface at baseline, 3 months, and 6 months after implant placement.

Statistical analysis

The mean and standard deviations (Mean ± SD) values were calculated for all clinical parameters, including PI, modified bleeding index (PBI), probing pocket depth (PPD), width of the keratinized gingiva (WKG), and radiographic crestal bone level. The mean data were analyzed for statistical significance by standard statistical method to compare data from baseline, 3 months, and 6 months for all the patients. Student’s paired t-test was used to compare baseline to those at 3 months and from 3 months to 6 months for all the patients. If the probability value (p) is more than >0.05, the difference observed was non-significant, and if the value is less than ≤0.05, it was considered significant.

## Results

**Figure 13:**
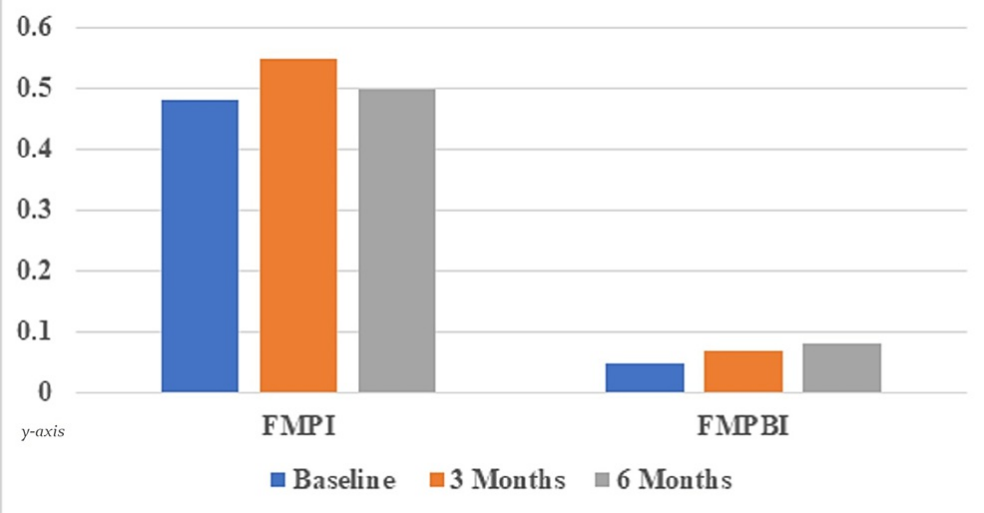
Graph depicting full mouth plaque index (FMPI) and full mouth papillary bleeding index (FMPBI) scores Comparison of full mouth plaque index (FMPI) and full mouth papillary bleeding index (FMPBI) scores at baseline, 3 months, and 6 months follow-ups.

 Twelve systemically healthy patients (seven females, five males) in the age group of 20-50 years (Mean age 29.64±10.65 years) received immediate implants in 14 fresh extraction sockets using a two-stage protocol. Out of 14 implants, nine were placed in the mandible (one in the single-rooted teeth, eight in multirooted teeth), and five were placed in the maxilla (five in single-rooted teeth) using a delayed loading protocol. The mean FMPI score at baseline was 0.48±0.22 mm, and at 3 months, it was 0.55±0.26 mm with a statistically non-significant mean difference of 0.07±0.11 mm. At 6-month follow-up, FMPI score was 0.5±0.31 mm, which was equivalent to the scores at 3 months with a statistically non-significant mean difference of 0.01±0.22 mm. At the 6-month evaluation, the mean FMPBI score during the study period remained low (<1) in all patients indicating satisfactory gingival condition throughout the study period. (Table 1; Figure 13).

**Table 1:** Depicting full mouth plaque index (FMPI) and full mouth papillary bleeding index (FMPBI) scores Comparison of full mouth plaque index (FMPI) and full mouth papillary bleeding index (FMPBI) scores at baseline, 3 months, and 6 months follow-ups (Mean ± SD; in mm)

Parameters	Baseline	3 months	Difference	p-value	6 months	Difference	p-value
FMPI	0.48±0.22	0.55±0.26	0.07±0.11	0.06	0.5±0.31	0.01±0.22	0.77
FMPBI	0.05±0.10	0.07±0.11	0.01±0.06	0.33	0.08±0.15	0.03±0.13	0.33

**Figure 14:**
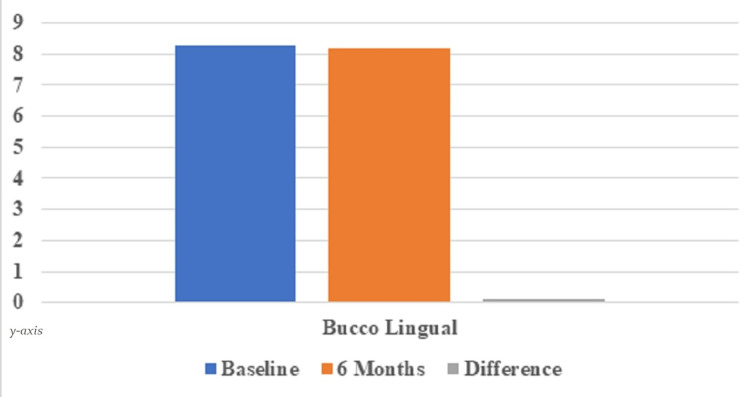
Graph depicting buccolingual dimension of socket immediately after extraction and at 6 months post-surgery Measurement of buccolingual dimension of socket immediately after extraction and at 6 months post-surgery after implant placement (Mean ± SD; in mm)

The mean horizontal gap distance, as measured intra-surgically, was 2.28±0.46 mm. The mean buccolingual measurement of the socket at baseline was 8.27±1.76 mm. Postoperatively, following 6 months of implant placement, the mean buccolingual measurement was 8.17±1.69 mm. When compared between mean measurements of the buccolingual dimension of the socket at the time of extraction and 6 months post-surgery, the mean bone loss was 0.10±0.09 mm, which was statistically non-significant (Table 2; Figure 14).

**Table 2:** Depicting buccolingual dimension of socket immediately after extraction and at 6 months post-surgery Measurement of the buccolingual dimension of socket immediately after extraction and at 6 months post-surgery after implant placement (Mean ± SD; in mm)

	At baseline	At 6 months	Differences (Mean bone loss)	p-value
Mean	8.27±1.76	8.17±1.69	0.10±0.09	0.87

**Figure 15:**
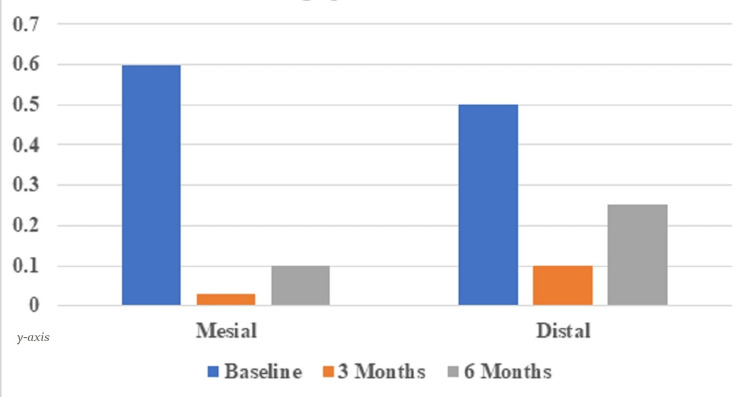
Graph depicting radiographic vertical bone level on mesial and distal surfaces of implant Mean radiographic vertical bone level on mesial and distal surfaces of implant at baseline, 3 months, and 6 months follow-up (Mean ± SD; in mm)

The mean radiographic vertical bone level at the mesial surface of the implant at baseline was 0.6±0.59 mm; at 3 months, it reduced to 0.03±0.13 mm with a statistically significant (p ≤ 0.05) mean bone gain of 0.57±0.64 mm. At 6 months, it was increased to 0.10±0.21 mm, with a mean bone loss of 0.5±0.67 mm, which was statistically significant (p ≤ 0.05). The baseline means a vertical bone level on the distal surface of the implant was 0.5±0.73 mm, and at 3 months, it was increased to 0.10±0.21 mm with a statistically significant (p ≤ 0.05) mean bone loss of 0.39±0.73 mm. At 6 months, it increased to 0.25±0.21 mm, with a mean bone loss of 0.25±0.70 mm, which was statistically non-significant (p ≥ 0.05) (Table 3; Figure 15).

**Table 3:** Depicting radiographic vertical bone level on mesial and distal surfaces of implant Mean radiographic vertical bone level on mesial and distal surfaces of implant at baseline, 3 months, and 6 months follow-ups (Mean ± SD; in mm)

Surface	The vertical distance between the shoulder of the implant and the most coronal bone to implant contact						
	At baseline	At 3 months	Differences (Mean bone loss)	p-value	At 6 months	Differences (Mean bone loss)	p-value
Mesial	0.6±0.59	0.03±0.13	0.57±0.64	0.005	0.10±0.21	0.5±0.67	0.01
Distal	0.5±0.73	0.10±0.21	0.39±0.73	0.05	0.25±0.21	0.25±0.70	0.20

**Figure 16:**
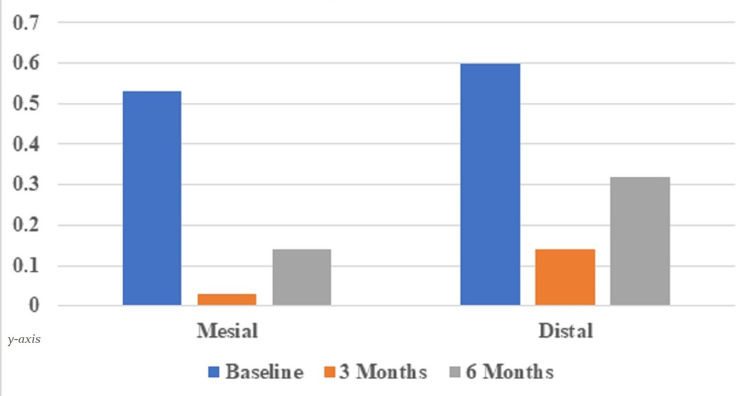
Graph depicting radiographic horizontal distance between the implant and the crest of the alveolar bone at baseline and 6 months follow-up Mean radiographic horizontal distance between the implant and the crest of the alveolar bone at baseline and 6 months follow-up (Mean ± SD; in mm)

At baseline, the mean radiographic HD between the implant and the crest of alveolar bone on the mesial surface was 0.53±0.41 mm. At 3 months, it reduced to 0.03±0.13 mm, with a statistically significant (p ≤ 0.05) mean bone gain of 0.5±0.42 mm. However, at 6 months, the mean radiographic HD on the mesial aspect increased to 0.14±0.23 mm, with a statistically significant (p ≤ 0.05) mean bone loss of 0.39±0.40 mm. The baseline means HD on the distal surface was 0.60±0.65 mm; at 3 months, it was decreased to 0.14±0.30 mm, with a mean bone gain of 0.46±0.45 mm. At 6 months, the HD increased to 0.32±0.31 mm on the distal surface with a mean bone loss of 0.28±0.46 mm, which was statistically significant (Table 4; Figure 16).

**Table 4:** Depicting radiographic horizontal distance between the implant and the crest of the alveolar bone Mean radiographic horizontal distance between the implant and the crest of the alveolar bone at baseline and 6 months follow-up (Mean ± SD; in mm)

Surface	The horizontal distance between shoulder of the implant and the most coronal bone to implant contact						
	At baseline	At 3 months	Differences (Mean bone gain)	p-value	At 6 months	Differences (Mean bone gain)	p-value
Mesial	0.53±0.41	0.03±0.13	0.5±0.42	0.001	0.14±0.23	0.39±0.40	0.002
Distal	0.60±0.65	0.14±0.30	0.46±0.45	0.002	0.32±0.31	0.28±0.46	0.04

Clinical parameters around the implant

Measurement of clinical parameters, including PPD and WKG around the implant. The WKG was measured at baseline and after 6 months post-surgery. At baseline, WKG was 3.21±0.69 mm, and at 6 months, it reduced to 2.92±0.61 mm with a statistical mean difference of 0.28±0.46 mm. PPD was measured around all the implants at 3 months and 6 months. At 3 months, PPD was 1.03±0.51 mm, and at 6 months, it increased to 0.44±0.50 mm with a statistically significant mean difference of 0.58±0.77 mm (Table 5).

**Table 5:** Depicting clinical parameters around implant Measurement of clinical parameters around implant at baseline and at 6 months (MV ± SD) WKG: width of keratinized gingiva; PPD: probing pocket depth

Parameter	Baseline	6 months	Difference	p-value
WKG	3.21±0.69	2.92±0.61	0.28±0.46	0.04
Parameter	3 months	6 months	Difference	p-value
PPD	1.03±0.51	0.44±0.50	0.58±0.77	0.01

Measurement of implant stability using CIMS at 3 months after definite prosthesis for all patients. At 6 months follow-up, all 14 implants showed a CIMS score of 0. Therefore, none of the patients dropped out of the study, with a 100% success rate of implant over a 6-months follow-up period.

**Figure 17:**
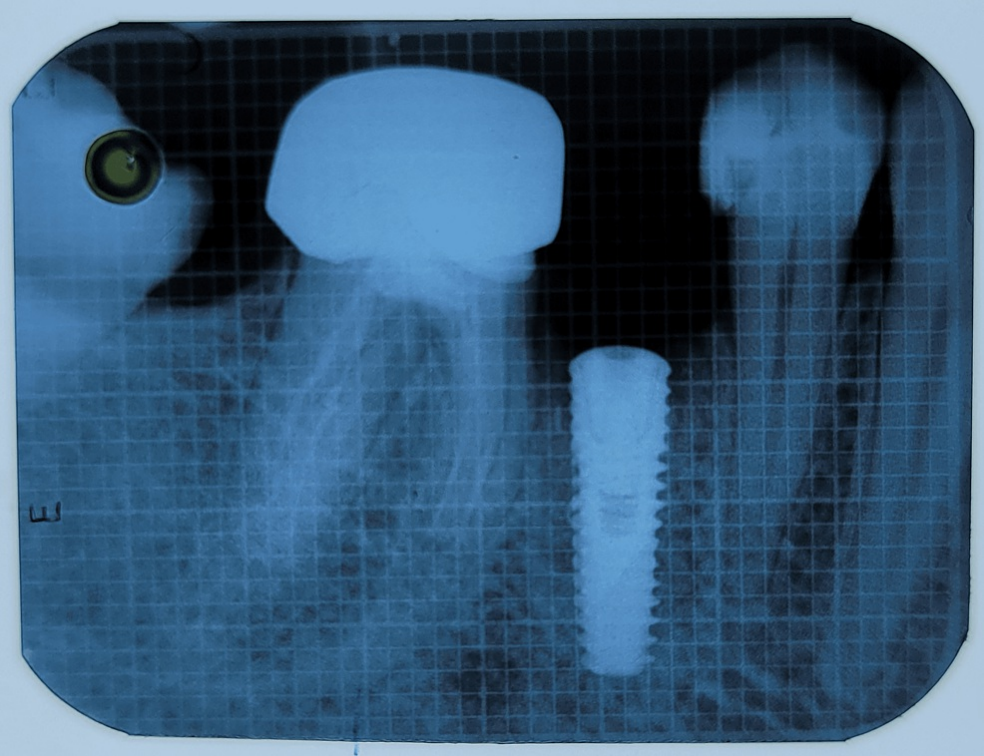
Depicting baseline radiograph obtained immediately postoperatively

**Figure 18:**
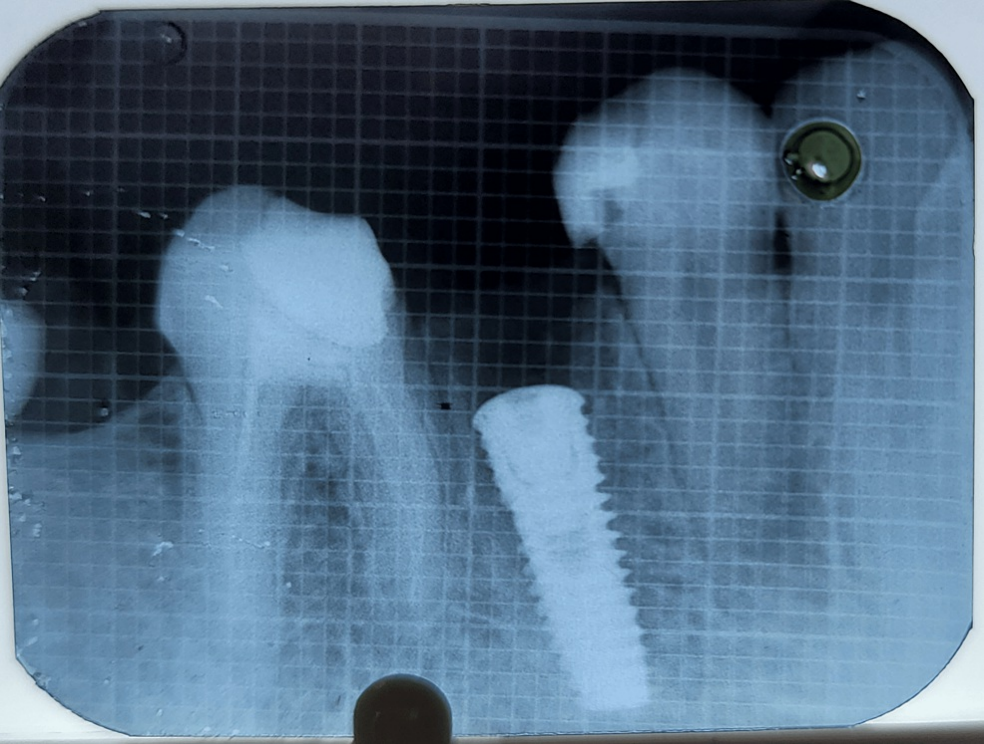
Depicting radiograph obtained at 3 months postoperatively

**Figure 19:**
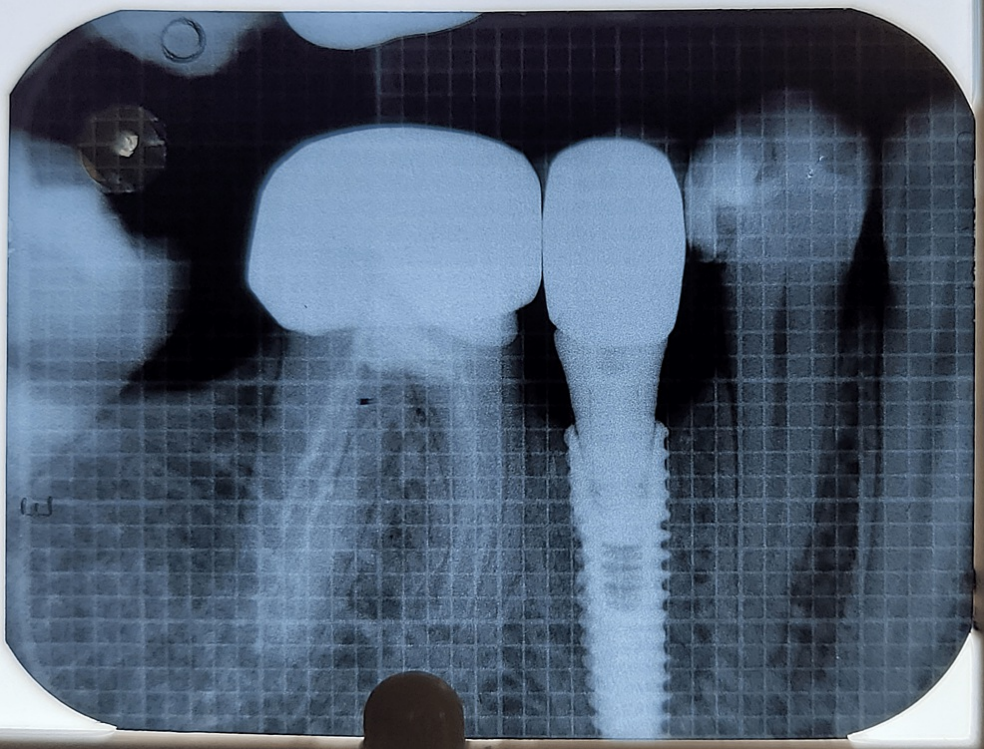
Depicting radiograph obtained at 6 months postoperatively

Figures 17-19 depict the radiographic results obtained at baseline, 3 months, and 6 months.

## Discussion

The width of the gap distance between the surface of the implant and the buccal bone during implant placement is critical for subsequent bone healing in a fresh extraction socket; as the gap broadens, the amount of BIC decreases, causing the apical shift of the highest BIC [26]. Following that, it was agreed that gap sizes of more than 1.5-2 mm needed the use of allograft or xenograft bone particles protected by a membrane to prevent soft tissue in-growth [12]. The implants with a gap distance of >1.5-2 mm were included and were simultaneously grafted with DFDBA and PRFM after implant insertion.

At 6 months follow-up, the coronal bone remodeling detected on CBCT revealed a minimal (0.1 mm) narrowing of the alveolar ridge in a buccolingual direction, with a mean bone loss of 0.10+0.09, which was statistically non-significant (p>0.05). Simon et al. (2011) conducted guided bone regeneration (GBR) employing DFDBA and PRFM membrane in 21 patients. Wherein the mean width measured 3 mm apical to the crest was 6.47 mm at the time of implant placement [27]. After 4 months of healing, results demonstrated a statistically significant reduction of 0.32 mm (4.71%) which could be attributed to the use of DFDBA and PRFM in the residual socket gap. The effect of combining PRF and DFDBA in immediate implants inserted into extraction sockets with periapical infection was studied by Madikeri et al., in 91.7% of cases, there was no variation in buccal gingival level, and the interproximal gap was completely closed. At 12 months, the implant had a survival rate of 91.67% [23].

At baseline, 3 months, and 6 months, a radiographic study on IOPA revealed that the implants were well fixed in the bone, with horizontal alterations in the most coronal BIC on the mesial and distal surfaces of the implants. With a mean bone loss of 0.4 mm, the horizontal alveolar bone crest level on the medial surface was 0.03+0.13 at 3 months and 0.14+0.23 at 6 months following implant implantation. The distal bone level was 0.14+0.30 mm at 3 months and 0.32+0.31 mm at 6 months, with a mean bone loss of 0.3 mm, which was statistically significant. The vertical bone level on the mesial surface was 0.03+0.13 mm at 3 months and 0.10+0.21 mm at 6 months, with a mean substantial bone loss of 0.5 mm. While on the distal surface, it was 0.10+0.211 mm at 3 months and 0.25+0.21 mm at 6 months, with a non-significant mean bone loss of 0.2 mm. Rossi et al. (2013) found vertical alveolar crest resorption of around 1 mm surrounding the implant, with only 0.3 mm of resorption on the mesial surface 4 months after immediate implant insertion [28]. Cornelini et al. (2005) found 0.8 mm bone resorption in 10 maxillary implants placed in extraction sockets to replace anterior teeth and premolars, while Chaushu et al. (2001) found 0.5 mm bone resorption after a year of immediately inserted and loaded implant [29,30].

The selection of grafting material for ridge preservation treatments is an important topic of discussion in implant dentistry. Autografts, allografts, xenografts, alloplasts, bioactive agents, or a mixture (composite) of grafts are among the graft materials available. For grafting an extraction socket, an autogenous bone graft is the most predictable and effective treatment option. Autogenous bone as a transplant source has a great capacity for osteogenesis and an ideal ability to integrate without immunologic complications [31-35]. Despite the fact that autogenous bone grafts are the gold standard, a variety of bone graft materials are now being employed successfully. It has been observed that employing DFDBA for grafting extraction sockets resulted in a rise in the number of new bone trabeculae as well as the average size of new bone trabeculae at all time intervals [36]. This could be due to DFDBA particles’ osteoconductive and osteoinductive properties. The action of bone inductive proteins called BMPs revealed during the demineralization process was thought to cause DFDBA to induce bone formation. The BMPs are involved in a biologic cascade that includes chemotaxis and matrix attachment, cell proliferation, and differentiation into cartilage, bone, and marrow [37]. It displays a rapid osteoinductive activity [38]. In principle, the clinical advantages of allografts are their equivalent mechanical and handling properties compared to autologous bone, may show the presence of collagenous matrix and proteins found in natural bone, despite the lack of vital cells, shorter surgical time required for implantation, as well as their easy accessibility, makes it a material of choice for a wide variety of applications [19].

Recently, a novel platelet concentrate known as PRFM has been studied in periodontal regenerative procedures. It’s a first-generation platelet concentrate with identical attributes to traditional PRP but better mechanical and handling properties. Its alpha granules possess growth factors that affect all the cells and tissue formation in the wound healing process via signaling transduction mechanisms, thus showing a huge potential significance in regenerative therapy [20,39]. In vitro, O'Connell et al. showed that live platelets in PRFM secreted six growth factors in almost the same concentration throughout the entirety of their trial, which lasted 7 days. (PDGF, VEGF, EGF, FGF, TGF and IGF) [40]. In combination with the bone graft, growth factors from the fibrin matrix over the initial 7 days prolongs the chemotactic properties and promote proliferation of both fibroblast and osteoblast, including extracellular matrix deposition, differentiation of mesenchymal cell, vascular proliferation, and deposition of extracellular matrix [22,41]. PRF traps circulating stem cells, leading to faster repair of large osseous lesions where stem cells are developing into osteoblasts [22]. When BSM, such as DFDBA, is combined with PRF, these two unique wound healing processes can occur at the same time. Their combination may accelerate bone healing and boost the graft material’s biological activity [42]. The use of PRF aids in the retention of bone graft material within the socket walls and the arrest of bleeding, as it is a fibrin clot [43]. PRF fibrin fragments act as a biological link between bone fragments thus assisting in further healing of the defect.

Within the scope of this study, a few limitations were noted including the small sample size that has limited statistical power and ethical considerations as well as associated patient non-acceptance have restricted the histological evaluation to assess the actual amount of bone regeneration. Moreover, in the context of this study further long-term controlled clinical trials are needed to confirm the findings.

## Conclusions

Immediate implant placement has the following advantages over conventional placement protocols: i.e., it aids in preventing bone resorption and socket remodeling that would otherwise occur; it preserves alveolar bone integrity and anatomy; it allows ideal positioning of the implant with favorable load distribution; it reduces treatment duration and number of surgical procedures; maintenance of soft tissue contours and height in aesthetic zones, and it improves patient acceptance of the treatment plan. Different techniques have been attempted to combat residual ridge contraction, the majority of which involved a mix of promptly inserted implants and the use of numerous graft materials and barrier membranes. Immediate implantation regenerative methods have been shown to minimize horizontal bone resorption changes in the buccal bone after immediate implant placement. Standardized treatment approaches might be explored to enable good and predictable long-term functional outcomes in alveolar bone regeneration and implant rehabilitation.
